# Evaluation of Heart Failure Apps to Promote Self-Care: Systematic App Search

**DOI:** 10.2196/13173

**Published:** 2019-11-11

**Authors:** Sahr Wali, Catherine Demers, Hiba Shah, Huda Wali, Delphine Lim, Nirav Naik, Ahmad Ghany, Ayushi Vispute, Maya Wali, Karim Keshavjee

**Affiliations:** 1 Centre for Global eHealth Innovation Techna Institute University Health Network Toronto, ON Canada; 2 Institute of Health Policy, Management and Evaluation Dalla Lana School of Public Health University of Toronto Toronto, ON Canada; 3 Department of Medicine McMaster University Hamilton, ON Canada; 4 Department of Health Research Methods, Evidence and Impact McMaster University Hamilton, ON Canada; 5 School of Pharmacy University of Waterloo Kitchener, ON Canada; 6 Centennial College Toronto, ON Canada; 7 InfoClin Inc Toronto, ON Canada

**Keywords:** mHealth, heart failure, self-care, mobile phone

## Abstract

**Background:**

Heart failure (HF) is a chronic disease that affects over 1% of Canadians and at least 26 million people worldwide. With the continued rise in disease prevalence and an aging population, HF-related costs are expected to create a significant economic burden. Many mobile health (mHealth) apps have been developed to help support patients’ self-care in the home setting, but it is unclear if they are suited to the needs or capabilities of older adults.

**Objective:**

This study aimed to identify HF apps and evaluate whether they met the criteria for optimal HF self-care.

**Methods:**

We conducted a systematic search of all apps available exclusively for HF self-care across Google Play and the App Store. We then evaluated the apps according to a list of 25 major functions pivotal to promoting HF self-care for older adults.

**Results:**

A total of 74 apps for HF self-care were identified, but only 21 apps were listed as being both HF and self-care specific. None of the apps had all 25 of the listed features for an adequate HF self-care app, and only 41% (31/74) apps had the key weight management feature present. HF Storylines received the highest functionality score (18/25, 72%).

**Conclusions:**

Our findings suggest that currently available apps are not adequate for use by older adults with HF. This highlights the need for mHealth apps to refine their development process so that user needs and capabilities are identified during the design stage to ensure the usability of the app.

## Introduction

Heart failure (HF) is the most important cardiovascular condition leading to hospitalization and rehospitalization in older adults, and it has a significant economic burden [1]. Despite an overall decline in HF hospitalization rates, readmission rates remain high [2,3]. A systematic review found that HF readmissions can be reduced if patients with HF adopt self-care (hazard ratio [HR] 0.80; 95% CI 0.71-0.89) [3,4]. Specifically, weight monitoring has been identified as a pivotal component of HF self-care as weight gain has been independently associated with a poor postdischarge prognosis, considering it is the last common step before worsening of clinical outcomes (HR per kg increase 1.16; 95% CI 1.09-1.23; *P*<.001) [5]. However, many older adults find daily weight monitoring and adjusting diuretics to be challenging [6-8]. In addition to managing comorbid conditions, many older adults with HF exhibit mild cognitive impairment and poor medication adherence, both of which are associated with reduced ability to self-care [9-11]. To adequately promote HF self-care, strategies should be targeted to the patient’s cognitive capabilities, learning needs, and literacy and numeracy levels [11].

Mobile health (mHealth) apps have been developed to support patients with self-care [12,13]. Unfortunately, although the initial uptake of mHealth apps looked promising, the majority of individuals have stopped using them because of reasons such as a loss of interest, manual data entry burden, and hidden costs [14,15]. Older adults do not commonly use mHealth apps because of the perception that they are not suited to their needs or capabilities [16]. This may explain the shortcomings of previous programs that have failed to promote self-care and utilize the opportunity to help decrease HF-related hospitalizations, deaths, and costs to health care systems [9-12].

Previous studies have reviewed current apps for HF self-care and found that there are limited number of apps available to support disease management [12,17]. Nevertheless, these studies were unable to effectively evaluate app quality because of their lack of disease specificity within the rating scale design [18,19]. For example, the commonly used Mobile Application Rating Scale (MARS) was able to provide an overall assessment of the quality of apps with respect to engagement, functionality, aesthetics, information, and subjective opinion, but it does not evaluate the usability or effectiveness of the app features specific for the disease population [12,15,18,19]. Therefore, generic health apps (eg, WebMD) receive higher app quality scores using the MARS even if they do not have crucial app features (eg, weight management) for proper self-care [17]. The lack of disease specificity can be attributed to the absence of a reference architecture to guide the scale’s development. Other chronic disease app rating scales or checklists have been developed using similar constructs as the MARS, leaving them to face the same shortcomings with their app evaluations [20,21]. This highlights the need to further evaluate the adequacy of the current HF self-care apps available.

To address this gap, we conducted a systematic search of all the apps currently available exclusively for HF self-care. We used Chindalo et al’s peer-reviewed mHealth app reference architecture to define the app design requirements [19]. Contrary to other rating scales, this architecture allows us to combine the evaluative components related to the aesthetics, usability, and HF self-care to effectively evaluate whether the current HF apps are meeting the end user’s self-care needs and capabilities [19]. The objective of this study was to determine the number of HF apps available and evaluate whether they met the criteria to promote HF self-care.

## Methods

### Search Strategy

We conducted an extensive search across Google Play and the App Store to identify all available apps for HF self-care. The search was facilitated with the use of following key terms: HF management, HF manager, HF self-care, HF, and HF tracker. Apps were included in the review if they (1) were HF specific and (2) contained a self-care component (ie, medication, symptom management, reminder system, and behavior tracking). Apps were excluded if they were intended for use in a conference, for education, or for reference purposes.

### App Adequacy Assessment

In accordance with Chindalo et al’s reference architecture, we developed a list of 25 major functions that would promote HF self-care for older adults [19,22-24] (Table 1). These features were identified according to HF self-care and patient-engagement guidelines [19] as well as the expertise from our clinician authors (CD and KK). Before the start of the study, a design session was conducted where CD (cardiologist/HF specialist) and KK (family physician/clinical information technology architect) created individual lists for potential app features. A second design session was conducted with CD, KK, and SW to finalize the list of app functions as well as the specific functions required for app adequacy. All 25 major functions were not deemed *required* to adequately promote HF self-care but were more beneficial if included. App adequacy was determined if the following standard disease management features were included: (1) diagnosis, (2) weight, (3) behavior tracking, (4) self-care, and (5) notifications. These 5 features were chosen based on their ability to capture factors related to HF management protocol, personalized care for older adults, and health promotion [25,26].

**Figure 1:**
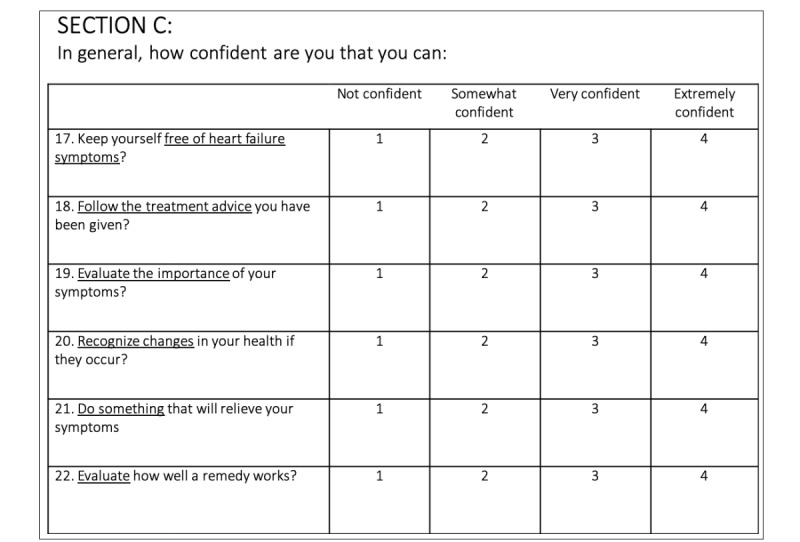
Sample heart failure self-care confidence questions.

Within each of the 25 functions, a list of descriptors was developed to help specify the components within the listed function. If the app included 1 of the descriptors, the feature was listed as present (Table 1). For example, the self-care feature consisted of 3 components including self-maintenance, self-management, and self-confidence [6,7]. Self-maintenance includes actions associated with treatment adherence, such as taking medication or following treatment regimens. Self-management includes the recognition of and response to changes in symptoms. Finally, self-confidence refers to the individual’s assurance in implementing necessary decisions during the management process. Self-confidence is not an explicit self-care behavior but has been recognized as an important moderator of self-care effectiveness [6,7,27]. Thus, self-confidence would not be captured in app functionality as clearly as self-maintenance and self-management, but instead could be expressed as a series of patient experience–related questions (Figure 1). Overall, if an app included any 1 of the 3 self-care components, the feature would be counted as being present.

**Table 1:** List of app features required for an adequate heart failure self-care app.

#	App feature	App descriptors
1	Prescribed	Physician prescribed for treatment; pharmacist recommendation
2	Diagnosis^a^	Patient predetermined diagnosis included (acute heart failure)
3	Patient demographics	Age; sex or gender; location
4	Patient sociocultural	Literacy; numeracy; socioeconomic status; culture/ethnicity; parental history
5	Patient symptoms	Shortness of breath; dizziness; orthopnea; leg edema or general swelling; paroxysmal nocturnal dyspnea
6	Patient behaviors	Smoking; exercise; fitness/movement; salt intake
7	Patient physiological observations	Heart rate; blood pressure; elevated jugular venous pressure; chest crackles; heart murmurs
8	Weight^a^	Management; monitoring; tracker
9	Comorbidities	Presence of other diseases (eg, diabetes and hypertension)
10	Drug list	List of medications
11	Laboratory results	Hemoglobin and hematocrit; creatinine and estimated glomerular filtration rate; brain natriuretic peptide; thyroid stimulating hormone; lipid profile
12	Diagnostic testing	Electrocardiogram; chest x-ray; echocardiogram
13	Behavior tracking^a^	Diet; exercise; patient-reported experience; compliance with medications
14	Education/recommendation	Behaviorally appropriate; culturally appropriate; health literacy appropriate; accredited/credible sources; evidence based
15	Self-care^a^	Self-maintenance; self-management: system provides patient with recommendation if clinical condition changes (eg, if weight increases, take extra Lasix); algorithm based or physician guidance; self-confidence
16	Health system utilization	Reviewed by family doctor; reviewed by nurse clinician, practitioner, or physician assistant; visit to ED^b^; hospitalization; seen by specialist
17	Notifications^a^	Presence of reminder or notification
18	Integrations	Integrated into personal health record and electronic medical record; integrated into other health and fitness apps
19	Social supports	Connect/share results with caregiver or family; contact caregiver or family
20	Patient reported outcome measure/patient reported experience measure	Patient experience of care; app experience; quality of life; cognitive assessment; patient progress
21	Incentives to use	Easy access to provider; gamification; social aspect—connect with others
22	Predictive analytics	Length of stay, acuity of admission, comorbidities, ED visits, (readmissions); hospital admission risk prediction
23	Outcomes	Visit to family physician, specialist, or ED; hospitalized; death
24	Safety issues	Risk of falls; worsening kidney function; hyper- or hypokalemia
25	User interface	Easy to navigate functionality; simple to screen with minimal content on each page; features for visual (font size and color), hearing (audio cues), or general accessibility

^a^Standard disease management feature for heart failure.

^b^ED: emergency department.

### App Screening and Evaluation

A total of 2 reviewers (SW and KK) completed a preliminary screening of apps available in both Google Play and the App Store using the key terms mentioned previously. Following the search, apps were reviewed according to their title and summary description (Figure 1). This screening served as a method to separate the bulk of non-HF self-care apps from further evaluation.

Once screening was completed, a calibration session was held among the 8 reviewers (SW, KK, AG, AD, HW, HS, NN, and DL). During the calibration session, each reviewer was asked to evaluate and score the same 5 apps before the start of the session (Figure 1). Reviewers had a Google Sheet created for them with the list of functions for evaluation. During the session, the 5 apps were then reviewed on a summary sheet, and any discrepancies regarding scoring were discussed and resolved. This allowed us to standardize the training among all reviewers and ensure that we had at least an 85% agreement rate when evaluating the remaining apps. Additional information and comments from the calibration session were recorded and added to the final protocol. The evaluation sheet on Google Sheets was also revised for the final app evaluations.

After the calibration session, the remaining apps were assigned for evaluation, where 2 different reviewers evaluated each app. Each evaluation was completed on the revised Google Sheet with the respective app assignments. Once all the app evaluations were completed, the data were combined into a summary sheet for review. Each app score was then reviewed by the 2 assigned reviewers to ensure that they were within the 85% agreement rate. To determine if the scores met the 85% agreement rate, we conducted interrater agreement statistic and reviewed the kappa value. If the apps did not meet the 85% agreement threshold, the 2 reviewers completed an in-person or virtual evaluation session to review the app discrepancies. Both reviewers were required to provide evidence (ie, screenshot or quote) to support the presence of the feature, and a discussion was held until consensus was achieved. All supporting evidence was sent to SW for a final review. Following consensus, a postreview screening was then completed to filter out any apps that were not HF or self-care specific. The remaining apps within the inclusion criteria were analyzed through a descriptive analysis to assess the app search’s findings.

Reviewers evaluated each app based on the description, screenshots, videos, and reviews available on each app store website. Owing to the limited resources and to ensure a consistent method of app evaluation, we did not download the apps. Our rationale for not downloading apps was also based on the premise that users decide to download an app after reviewing it externally [28-31]. Many of the guidelines assisting patients with choosing a health app have urged users to become more meticulous with the apps they install and, in turn, have provided them with a series of questions to consider before downloading [29-31]. For example, in 2 articles, they suggested users consider the following questions before downloading an app: (1) does the app consider your needs (ie, symptoms and disease management), (2) is the app made by a health care system/physician or by a controversial company (ie, pharmaceutical company), (3) does the app have positive reviews (ie, on the Web or by physicians), (4) is the app regularly updated, and (5) are the app features relevant for you (ie, review screenshots and description of features) [29,30]. Currently, about 90% of most information for decision making about app adoption is available in its documentation [29,32,33]. Given the minimal incremental information available from the app itself, we felt that downloading the apps would not significantly change our evaluation.

In accordance with the guidelines for app review before download, reviewers also extracted the following data from each app: number of downloads, date of last update, cost, and developer [29,30]. However, considering that apps from the App Store do not publicly list their number of downloads or date of last update, reviewers omitted the number of downloads criteria for these apps and used the latest version date for the last update.

### Reviewer Training

Each reviewer selected was equipped with postsecondary experience in the electronic health or health technology field to allow them to effectively evaluate the respective apps. Reviewers were required to follow the training protocol in accordance with the mHealth design architecture as well as attend the calibration session previously described [19].

## Results

**Figure 2:**
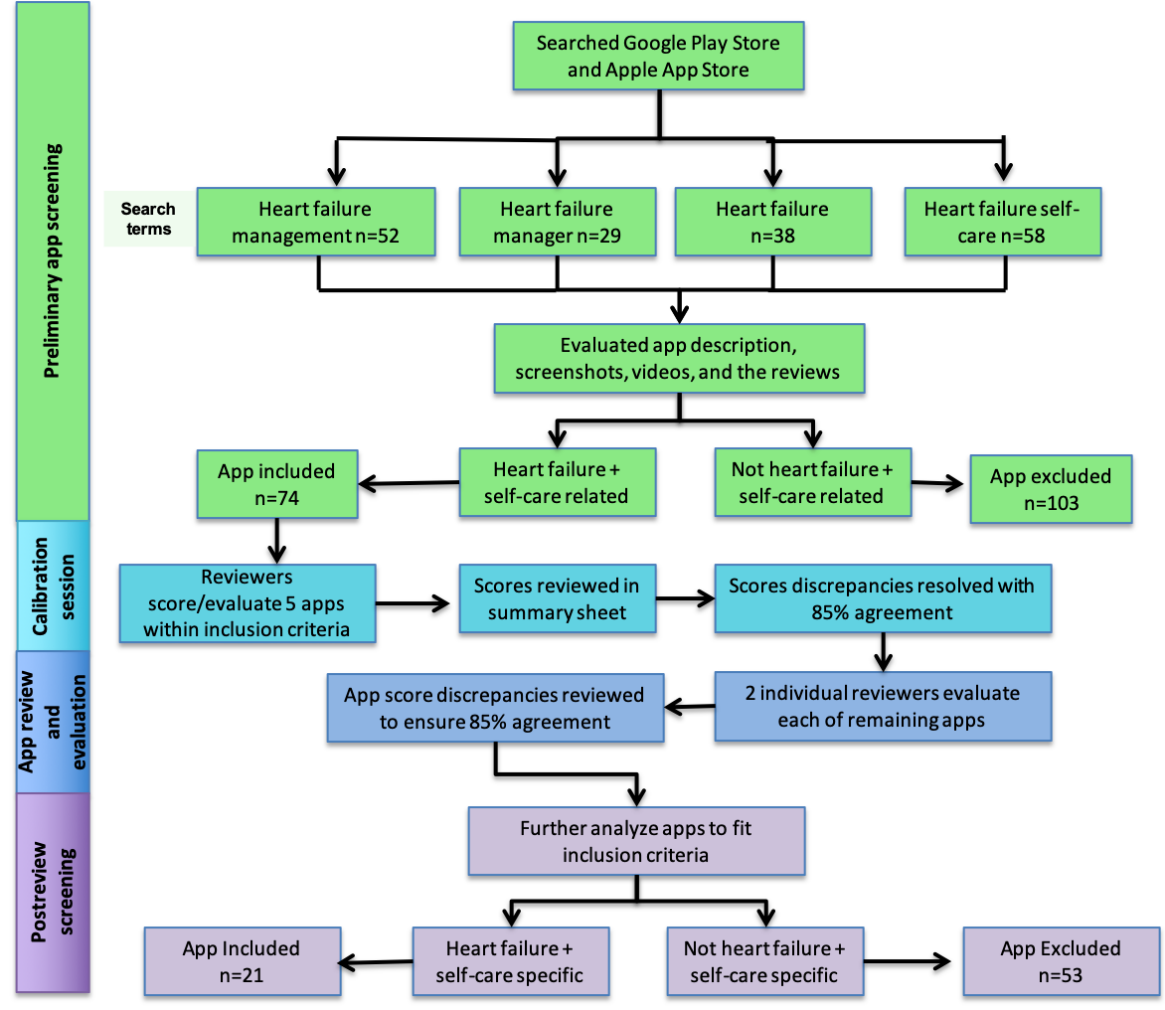
Conceptual study design of the mobile health app review.

Preliminary screening identified a total of 74 apps as HF self-care apps within the combined app store searches (Figure 2). From these 74 apps, none of the apps had all the 25 listed features required to promote HF self-care, and only 32% (24/74) apps had 10 features or more present. Moreover, only 51 out of the 74 apps had a self-care feature present. Instead, the majority of the apps reviewed were used mainly for education purposes (Table 2).

**Table 2:** Features present in the reviewed heart failure self-care apps (N=74).

App feature	Apps, n (%)
Education/recommendations	67 (90)
Self-care	51 (68)
User interface	51 (68)
Diagnosis	40 (54)
Notifications	33 (44)
Weight	31 (41)
Patient demographics	28 (37)
Patient symptoms	27 (36)
Patient physiological observations	26 (35)
Behavior tracking	22 (29)
Patient behaviors	19 (25)
Drug list	18 (24)
Patient reported outcome measure/patient reported experience measure	18 (24)
Incentives to use	18 (24)
Comorbidities	9 (12)
Lab results	9 (12)
Diagnostic testing	9 (12)
Social supports	9 (12)
Prescribed	8 (10)
Health system utilization	8 (10)
Outcomes	7 (9)
Predictive analytics	6 (8)
Patient socio-cultural	6 (8)
Safety issues	0 (0)

Following the postreview screening, 21 apps were listed as both HF and self-care specific. Moreover, 53 apps were excluded for the following reasons: (1) HF specific but not for self-care (n=9), (2) used for self-care but not specifically for HF (n=16), and (3) neither HF nor self-care specific but used for general cardiac education (n=28). As there is an increasing number of apps for entertainment or novelty purposes, from the 53 apps excluded, 12 were shortlisted for this reason. From the 21 HF self-care apps included, more than 50% (12/21, 57%) of the apps had 10 features or more (Table 3; Multimedia Appendix 1). The apps scores ranged from 0 to 18, where HF Storylines achieved the highest score. The most prevalent feature among these apps was diagnosis and user interface (20/21, 95%; Table 3). However, from the included apps, only 1 was found to have been evaluated by patients or investigated in a clinical setting (Medly). All reviewer’s app scores fell within the 85% agreement rate (mean kappa=0.86).

Many apps include features such as patient demographics, patient symptoms, education, self-care, notifications, and, most notably, weight. Unexpectedly, less than 50% (9/21) of the apps included a patient behavior or a behavior tracking feature, both of which are vital for adequate HF self-care (Table 3). Only 11 of the apps included a drug list within the app to keep track of medications or, more specifically, the usage of diuretics. In addition, a limited number of apps included social support (5) or were by prescription (4), and even fewer included a patient sociocultural (3) or comorbidities (2) feature. None of the reviewed apps included diagnostic testing, predictive analytics, outcomes, or safety measures (Table 3).

**Table 3:** Features present in filtered heart failure self-care apps (N=21).

App feature	Apps, n (%)
Diagnosis	20 (95)
User interface	20 (95)
Self-care	19 (90)
Notifications	18 (85)
Education/recommendations	17 (80)
Weight	17 (80)
Patient demographics	13 (61)
Patient symptoms	13 (61)
Patient physiological observations	11 (52)
Drug-list	11 (52)
Behavior tracking	10 (47)
Incentives to use	10 (47)
Patient behaviors	9 (42)
Integrations	8 (38)
Patient reported outcome measure/patient reported experience measure	8 (38)
Social supports	5 (23)
Health system utilization	4 (19)
Prescribed	4 (19)
Patient sociocultural	3 (14)
Lab results	3 (14)
Comorbidities	2 (9)
Diagnostic testing	0 (0)
Predictive analytics	0 (0)
Outcomes	0 (0)
Safety issues	0 (0)

Table 4 displays a list of the total score of the filtered HF self-care apps with their respective app characteristics. Of the apps available, only 9 listed their number of downloads, and among these apps, the number of downloads varied with the total app scores. Surprisingly, the lowest-scoring app had a higher number of downloads compared with the highest-scoring app. This discrepancy could be linked to more effective marketing strategies, public brand awareness, or a well-regarded national health organization for certain apps [31,32].

With respect to cost, the majority of apps could be downloaded for free; however, 2 apps had an associated cost. For consistency, apps were not downloaded. As a result, we found that the 2 apps with a download cost had relatively lower scores (score of 5=US $50 and score of 8=US $7) and did not list their number of downloads.

Each app’s last update varied from 2013 to 2019; however, most of the recently updated apps received higher scores. One of the most recently updated apps (HF Storylines) obtained the highest total app score of 18.

**Table 4:** Total score of the filtered heart failure self-care apps and their corresponding number of downloads, last updates, and cost (N=21).

App name	Total app score	Number of downloads	Last updated	Cost (US $)	Developer
Heart Failure	2	1000-5000	December 22, 2014	0	Leon Do
HF Coach	5	—^a^	April 29, 2016	50	Etectera Edutainment Inc
HF Defender	7	1000-5000	November 10, 2016	0	Cardio Fortress
HF Buddy	8	—	June 08, 2016	0	Singapore Health Services
HF Monitoring	8	—	February 22, 2017	0	Van Phuc Nguyen
HF Tracker	8	—	October 31, 2014	0	Rebecca Boxer
HF Path	8	—	January 04, 2017	7	American Heart Association
HF Log	9	—	February 09, 2016	0	Narnar LLC
HF Buddy	9	500-1000	April 08, 2016	0	—
Heart Scribe	10	50-100	July 30, 2016	0	Rohan Tanjea
Heart Failure Health	11	—	—	0	Self-Care Catalyst Inc
Health Plus	12	—	May 12, 2016	0	Hany Assaad
Heart Lessons	12	—	Apr 17, 2017	0	Palo Alto Medical Foundation for Health Care, Research and Education
My HF	12	1000-5000	September 22, 2016	0	Les Laboratoires Servier
WOW ME 200mg	12	100-500	July 24, 2013	0	AtantiCare Regional Medical Center Inc
HF Self- Management	13	—	August 11,2015	0	—
Pulsario	14	—	October 07, 2016	0	Cardio Fortress Inc
My Heart Mate	14	—	November 14, 2016	0	Elevator Entertainment
Heart Partner	16	—	October 07, 2016	0	Novartis Pharmaceuticals
Medly	17	—	Ongoing	—	University Health Network
HF Storylines	18	500-1000	March 10, 2017	0	HF Society of America

^a^Not available.

## Discussion

### Principal Findings

Self-care is pivotal for HF patients to prevent worsening of HF, yet the majority of current HF apps available are neither HF or self-care specific. To our knowledge, this is the first study to identify and evaluate apps exclusively for HF self-care. We found 21 apps that were both HF and self-care specific. From the 21 apps, few contained key features such as behavior tracking. Apps that included the self-care feature were also listed as only being capable of self-maintenance. Thus, patients would be able to, at most, follow their treatment regimen but would not be able to respond to any changes. Potential features to expand on self-care could include medication titration algorithms to adjust medication doses according to weight fluctuations or the use of telehealth services to connect with a physician to modify their treatment regime [34,35]. Loop diuretics are currently used as the agent of choice for reducing symptoms of HF and controlling weight. Diuretics are traditionally adjusted by physicians. However, with self-directed medication titration becoming more commonly used for chronic disease management, this feature could be an opportunity to improve patient HF self-care in the home setting [34,35].

Our findings suggest that the current available apps are not able to support patients adequately with HF self-care; instead, are in need of further redesign or development. Many developers may have limited resources to accommodate all 25 features in a single app. Therefore, to appropriately engage patients in self-care, apps should at minimum have the following functions: (1) diagnosis, (2) weight, (3) behavior tracking, (4) self-care, and (5) notifications. However, from our systematic search, none of the apps even had these 5 functional features. Not only are these app features key for HF self-care but they can also be easily transferable to other health conditions, such as diabetes or asthma, as it captures the essential components for treatment management. The specifics detailing each feature will differ depending on the condition, but it provides a sufficient baseline category to allow the consumer, researcher, or clinician to incorporate key components for the app evaluation. The consumer, researcher, or clinician may list similar or different components within the 5 features, but with this, they are able to incorporate their perspective within 5 wider categories, while maintaining its relevance for multiple audiences. A prime example of an effective mHealth app is BlueStar from WellDoc Diabetes Management. The BlueStar app is a digital therapeutic for diabetes mellitus type 2 that serves as a virtual coach for patients, providing tailored guidance and facilitating the coordination of diabetes care with their existing care team [36]. BlueStar is a clinically validated tool developed by endocrinologists and clinical diabetes educators and has been evaluated in a clinical trial and reviewed in over 40 publications [37,38]. Patients who used BlueStar showed significant improvements in their diabetes management and reported a high satisfaction when using the app. These improvements are strongly linked to the diabetes expertise leading the development and evaluation of this tool, as their guidance helps ensure that the intervention is aligned with self-management principles integral to patient care [36,37]. Older adults with chronic disease already face many challenges with managing their condition, and the use of technology may further contribute to their difficulties if poorly designed. To ensure apps assist with patient self-care regimens, they should be developed in a manner similar to BlueStar, where specific disease management and patient usability criteria are used to both design and evaluate app effectiveness [37].

One surprising finding from this app search was that the lowest-scoring apps had a relatively higher number of downloads compared with the highest-scoring app (Table 4). These findings ultimately question whether the inclusion of more app features or just the standard features for disease management are more appealing for the end user. Features that patients and consumers view as valuable can vary depending on their self-care abilities. However, many studies have indicated that the primary reason apps fail to maintain user activity is because of the complexity of the app as a whole or the lack of growth in app functionality in accordance with user needs [39,40]. Thus, as effective self-care promotion is the primary goal of app usage, apps would require the inclusion of the standard disease management features, but their presentation should also be modified to accommodate for the challenges older adults face with app use. In previous literature, older adults have indicated that in-app customizable considerations improved the likelihood of their continued usage as they were able to modify their preferences according to their changing needs [20,21]. This could include characteristics such as customizing screen or font sizes and incorporating text to speech, audio cues, or in-app automation features as needed [21,22].

Nevertheless, it is also important to note that the fact that lower-scoring apps had higher downloads can also be attributed to several external factors promoting public app awareness. This includes factors such as effective marketing, links to a national health body, or the use of Web-based search engine optimization [29,32]. From our findings, we found that the app with the highest downloads (My HF) had a moderate score of 12, but it was developed by a privately owned pharmaceutical company that specializes in medication for cardiological conditions (Les Laboratories Servier). Although apps built by health care systems scored the highest, they had much lower download rates (Table 4). With these discrepancies between app scores and the number of downloads, our findings display how higher downloads may not be an appropriate representation for app effectiveness because of its potential ties to a developer with more marketing power.

### Limitations

The limitations of our study were as follows: (1) the apps were not downloaded but were reviewed based on app description, screenshots, videos, and reviews from current/past users, and there are good reasons to believe that the quality of the assessment is not severely compromised, as mentioned above; (2) descriptions for review varied in detail and quality (eg, download data not available for Apple iOS apps); (3) we were only able to review the number of downloads but could not quantify active app use; and (4) actual HF patients were not consulted to define the criteria for adequate HF self-care app.

### Future Research

Future studies should involve end users to better understand their needs with the design of an app to ensure the uptake and usability of an intervention. Specifically, the use of engagement strategies with HF patients and health care providers would be strongly desirable to ensure the findings of this study are congruent with what is experienced in reality. In our app evaluation, we incorporated app user reviews to assess user perspectives, but this method is limited to the feedback available and the quality of the responses on the Web. This study also did not evaluate the value of the 5 functional criteria for HF self-care compared with the remaining categories. Future studies should aim to understand the relative importance of each criteria in relation to patient outcomes, potentially with the use of focus groups or user testing to develop priority weightings for each function. In addition to this, as the potential rise in scale modification for disease specificity could lead to inappropriate features being selected for app shortlisting, there is a need to also evaluate the priority features in relation to other common chronic conditions (ie, diabetes and asthma). We believe there is value in the inclusion of the 5 minimum features for app evaluation, as it allows for specific components to be embedded within wider priority categories and provides a baseline mode to manage the use of multiple modified scales. However, before any scales can be managed or reviewed, future studies need to confirm the reliability of the 5 features for the management of priority app categories.

### Conclusions

In summary, our study was the first to specifically evaluate HF self-care apps according to the criteria essential to promote HF self-care for older adults. We found that there was a lack of usable apps to promote HF self-care for older adults, and this is mainly because of the lack of a patient-centered design. With a rise in the aging population, identifying features pivotal for patient self-care will be crucial to increase their user experience and ensure the longevity of the app’s use.

## Appendix

Multimedia Appendix 1 Total features present in preliminary and postreview screening apps. PROMS: patient reported outcome measure; PREMS: patient reported experience measure.

## Abbreviations

HF: heart failure
HR: hazard ratio
mHealth: mobile health
MARS: Mobile Application Rating Scale
