# Supplementary material for: Evaluation of Heart Failure Apps to Promote Self-Care: Systematic App Search
Source: JMIR Mhealth Uhealth. 2019 Nov 11;7(11):e13173. doi: 10.2196/13173 (PMC6878098; doi:10.2196/13173)

Multimedia Appendix 1. Total features present in preliminary and postreview screening apps. (Patient Reported Outcome Measure (PROMS), Patient Reported Experience Measure (PREMS))

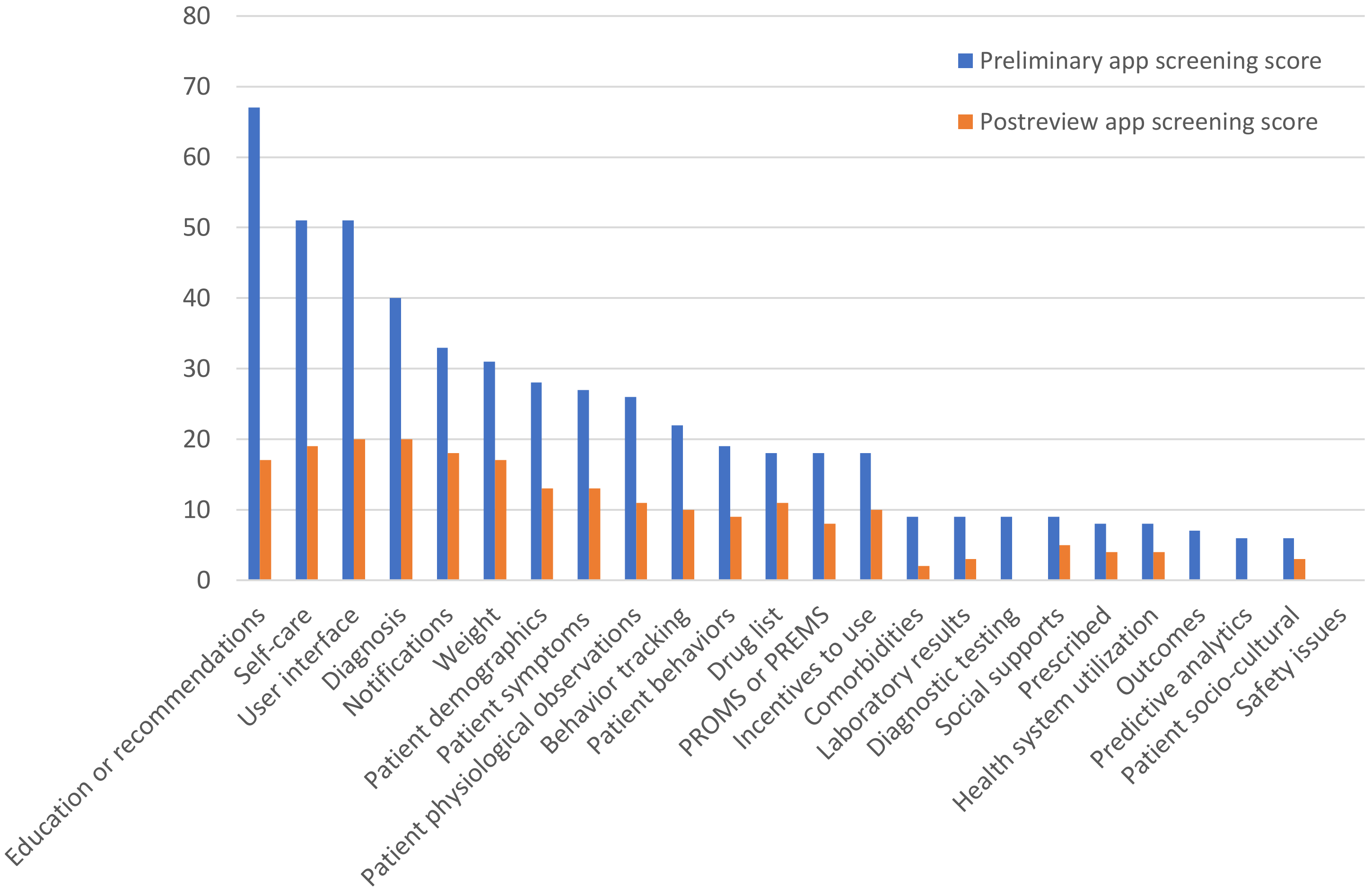

Supplement: Multimedia Appendix 1 [file mhealth_v7i11e13173_app1.pdf]
